# Retraction of “XRCC1 and hOGG1 polymorphisms and endometrial carcinoma: A meta-analysis”

**DOI:** 10.1515/med-2024-1000

**Published:** 2024-06-12

**Authors:** Shengke He, Xiujuan Zhao, Ruifang Mu, Zhongjun Pan, Jinglan Mai

**Affiliations:** Department of Pathology, Danzhou People’s Hospital, Nada Town, Danzhou, Hainan, 571799, China; Department of Gynaecology and Obstetrics, Danzhou People’s Hospital, Nada Town, Danzhou, Hainan, 571799, China; Occupational Physical Examination Outpatient, Haikou Center for Disease Control and Prevention, No. 56 Yehai Avenue, Qiongshan District, Haikou, Hainan, 570203, China

Retraction of: He S, Zhao X, Mu R, Pan Z, Mai J. XRCC1 and hOGG1 polymorphisms and endometrial carcinoma: A meta-analysis. Open Medicine 2024;19(1):20240913, DOI: 10.1515/med-2024-0913.

This article has been retracted by agreement between the authors, the journal Editor in Chief, Professor Vittorio Calabrese, and Walter De Gruyter GMBH. The retraction has been agreed at the request of the corresponding author Dr. Jinglan Mai, due to a conflict of interest between the first two authors. The retraction has been approved by all co-authors.

